# The effects of functional foods mixture on inflammatory cytokines and biochemical findings in hospitalized patients with COVID-19: a randomized double-blind controlled trial

**DOI:** 10.1186/s13063-023-07481-z

**Published:** 2023-07-05

**Authors:** Hadi Esmaeili Gouvarchinghaleh, Fateme Kiany, Karim Parastouei, Gholamhosein Alishiri, Nematollah Jonaidi Jafari, Abbas Ali Imani Fooladi, Afsaneh Pargar, Ali Ghazvini, Reza Mirnejad, Mehdi Raei, Ahmadreza Sharifi Olounabadi, Mansour Babaei, Soleyman Heydari, Hosein Rostami, Alireza Shahriary, Gholamreza Farnoosh, Vahid Sobhani, Mohammad Mahdi Mazhari, Farshad NajjarAsiabani

**Affiliations:** 1 Applied Virology Research Center, Baqiyatallah University of Medical Sciences, Tehran, Iran; 2 Department of Nutrition, School of Allied Medical Sciences, Ahvaz Jundishapur University of Medical Science, Ahvaz, Iran; 3 Health Research Center, Life Style Institute, Baqiyatallah University of Medical Sciences, Tehran, Iran; 4 Chemical Injuries Research Center, Systems Biology and Poisonings Institute, Baqiyatallah University of Medical Sciences, Tehran, Iran; 5 Applied Microbiology Research Center, Systems Biology and Poisonings Institute, Baqiyatallah University of Medical Sciences, Tehran, Iran; 6 Trauma Research Center, Baqiyatallah University of Medical Sciences, Tehran, Iran; 7 Molecular Biology Research Center, Systems Biology and Poisonings Institute, Baqiyatallah University of Medical Sciences, Tehran, Iran; 8 Medicine, Quran and Hadith Research Center, Baqiyatallah University of Medical Sciences, Tehran, Iran; 9 Health Management Research Center, Baqiyatallah University of Medical Sciences, Tehran, Iran; 10 Applied Biotechnology Research Center, Baqiyatallah University of Medical Sciences, Tehran, Iran; 11 Exercise Physiology Research Center, Life Style Institute, Baqiyatallah University of Medical Sciences, Tehran, Iran

**Keywords:** COVID-19, Functional food, Randomized controlled trial, Inflammation

## Abstract

**Background:**

The coronavirus disease 2019 (COVID-19) pandemic has been shown to affect nutritional recommendations. Some functional foods have been demonstrated to be useful in the treatment of people with COVID-19. However, little is known about the impact of combining functional foods on disease control. This study aimed to investigate the effects of functional foods mixture on serum levels of inflammatory cytokines and biochemical findings in patients with COVID-19.

**Methods:**

A randomized double-blind controlled trial was conducted in Baqiyatallah Al-Azam hospital in Tehran, Iran. Sixty patients were randomly assigned to receive either a soup containing functional foods (*n* = 30) or a usual soup (control group) (*n* = 30). Participants’ sociodemographic information was gathered using a general questionnaire. Blood levels of inflammatory markers and biochemical findings were assessed using standard protocols.

**Results:**

The results showed that soup containing functional foods was more effective in controlling serum levels of D-dimer, blood urea nitrogen, and creatinine than the control group (*P* < 0.05). Also, more significant improvement was found in the intervention group vs control group in terms of interleukin (IL)-1β, IL-6, IL-17, IL-10, and tumor necrose factor-α (*P* < 0.05). In contrast, the control intervention more efficiently controlled potassium levels and reduced quantitative C-reactive protein than the intervention group (*P* < 0.05).

**Conclusions:**

This study indicates a soup containing functional foods could alleviate biomarkers of inflammation in patients with COVID-19. However, its effectiveness on biochemical findings remained inconclusive which warranted further research.

**Trial registration:**

IRCT, IRCT20180201038585N11. Registered 23 August 2021, https://www.irct.ir/trial/57338

## Introduction

The World Health Organization (WHO) declared coronavirus disease 2019 (COVID-19) an international public health emergency on January 30, 2020, following the discovery of a new coronavirus in pneumonia patients [1]. There are a variety of clinical symptoms associated with the disease, including fever, cough, fatigue, acute respiratory distress syndrome, anorexia, and dyspnea [2], while increased C-reactive protein, decreased albumin levels, increased interleukin-6 levels, increased erythrocyte sedimentation rate, decreased eosinophils, lymphopenia, and increased lactate dehydrogenase are the most common laboratory findings [3]. In addition, evidence suggests that inflammation associated with the viral infection and associated cytokine storms, especially in severe cases, play an important role in the development of symptoms in COVID-19 patients [4, 5]. Besides, it has been shown that the coexistence of chronic noncommunicable diseases and COVID-19 can intensify inflammatory processes and even lead to the death of the patient [6].

Vaccination, wearing a mask, washing hands regularly with soap, disinfecting surfaces, avoiding crowds, and washing hands regularly with soap are all effective ways to prevent this illness [7–9]. However, studies showed that following a healthy eating pattern can help improve the immune system and prevent severe infections [10–12]. There is evidence that optimal nutritional status modulates inflammatory processes and the immune system’s ability to respond to regular oxidative stress [13]. The most important nutritional factors with anti-inflammatory properties include omega-3 fatty acids [14], vitamin A [15], and vitamin C [16]. Also, a variety of phytochemicals, including polyphenols [17], and carotenoids [18] that are found mainly in plant-based foods showed to have anti-inflammatory properties. Fermentation of dietary fiber in the gastrointestinal tract and production of short-chain fatty acids also play an important role in the anti-inflammatory activities of a plant-based diet [19].

Consumers’ needs for food production have changed significantly over the past decades, and new perspectives on the impact of nutrients on physiological function and health have led to the development of functional foods [20]. In fact, a food product can be considered functional if in addition to the nutritional role of food, it has beneficial effects on the function of one or more human organisms, improves mental health, or reduces the risk of disease [21]. Based on previous evidence, the most important beneficial effects of consuming functional foods include the following: reducing the risk of cancer, improving heart health, strengthening the immune system, improving gastrointestinal health, protecting urinary tract health, anti-inflammatory effects, lowering blood pressure, eye protection, antibacterial and antiviral activity, and reducing osteoporosis and anti-obesity effects [22]. Interestingly, few studies have also shown protective effects of functional foods, such as probiotics [23–25] and fish oil [26], and nutraceuticals derived from functional foods such as curcumin [27, 28] and quercetin [29] against complications associated with COVID-19. Nevertheless, to date, no study has examined the effects of combining natural functional foods on complications caused by COVID-19. Therefore, the purpose of this study was to determine whether a combination of functional foods would affect inflammatory cytokines and laboratory findings in patients with COVID-19.

## Methods and materials

### Study design

The study was a 7-day randomized double-blind controlled trial (IRCT20180201038585N11). Participants were randomized to receive either a soup containing functional foods (*N* = 30) or a barley soup as control (*N* = 30). Randomization was performed by a fourth block randomization schedule provided by random numbers table. Identical sealed packages were used to conceal the interventions. The soups were given to the participants for 7 consecutive days. Blood samples were collected at the beginning of the study and after 7 days to measure inflammatory cytokines as well as laboratory findings.

### Study population

The study participants were patients with COVID-19 who were admitted to Baqiyatallah Al-Azam hospital in Tehran, Iran, from September 20, 2021, to January 19, 2022. Patients of both sexes aged 20–70 years who were diagnosed with COVID-19 were enrolled in this study. Diagnosis with COVID-19 was performed by polymerase chain reaction (PCR), clinical chest tomography (CT scan), decreased oxygen saturation capacity to less than 93%, decreased systolic blood pressure to less than 100 mmHg, or a drop of more than 30 mmHg from its previous value, fever, cough, and increased blood levels of CRP or ESR, which showed infection with COVID-19. People on special diets, pregnant or lactating women, and patients with special diseases (HIV, cancer, patients undergoing chemotherapy, immunized patients, patients in need of hospitalization in the intensive care unit, and patients with uncontrolled heart, kidney, and liver diseases) were not eligible to participate in the study. Participants were excluded if they refused to continue the study or if their illnesses worsened. The study protocol was approved by the ethics scientific committee of the University of Baqiyatallah (ethical code number: IR.BMSU.REC.1399.149), and all participants filled in an informed consent form before participating in the study.

### Intervention groups

In this study, the intervention group received a soup containing 30 g of functional foods (peeled wheat, rice, mung, pea, apple, quince, carrot, cowpea, almond, onion, fresh garlic, parsley, coriander, leeks, mint, pennyroyal, spinach, beets, ground celery seeds, alfalfa sweat, yarrow sweat, chamomile sweat, olive oil, roasted sesame, roasted black seed, and roasted turmeric), and the control group received a barley soup for 7 consecutive days. Before the intervention, the soups were provided by the Amadeh Laziz Co. and packaged identically. On intervention day, the soups were given to the participants at 10 am as a snack before lunch.

### Socio-demographics and anthropometric measurements

A general questionnaire was used to collect socio-demographic information including age, gender, marital status, education level, disease history, and main clinical symptoms of COVID-19 disease. Weight and height were also measured, and BMI was calculated as (weight (kg)/ (height (m))2).

### Evaluation of laboratory findings

The results of biochemical and laboratory tests of participants were obtained from hospital databases, which evaluated them at the beginning and end of the intervention after a 12-h fasting period. These laboratory findings included the values for D-dimer (mg/l), blood urea nitrogen (BUN) (mg/dl), creatinine (Cr) (mg/dl), serum glutamic-oxaloacetic transaminase (SGOT) (u/l), serum glutamic-pyruvic transaminase (SGPT) (u/l), lactate dehydrogenase (u/l), sodium (Na) (mEq/l), and potassium (K) (mEq/l).

### Evaluation of inflammatory cytokines

The patient’s blood samples were collected at the beginning and end of the intervention after 12-h daytime fasting. Afterward, the serums were allocated and stored at − 80 °C until the day of analysis. Serum cytokine levels including interleukin-1 beta (IL-1β) (pg/ml), interleukin-6 (IL-6) (pg/ml), interleukin-17 (IL-17) (pg/ml), interleukin-10 (IL-10) (pg/ml), quantitative C-reactive-protein (CRP-q) (mg/l), tumor necrosis factor-alpha (TNF-a) (pg/ml), and interferon-gamma (IFN-γ) (pg/ml) were measured using an Abcam ELISA kits according to the manufacturer’s instructions, and the values were reported as pg/ml for each cytokine.

### Statistical analysis

Kolmogorov–Smirnov was used to evaluate the normal distribution of quantitative variables. Independent sample *t*-test was used for comparison of quantitative variables, and chi-square or Fisher’s exact tests were used for comparison of qualitative variables between the two groups. Analysis of covariance (ANCOVA) was used for comparisons between the two groups post-intervention after adjusting for baseline values and other confounders such as age, sex, and BMI. Paired *t*-test was used to compare within group difference from baseline to post-intervention period. The data were analyzed using the SPSS software (version 24, SPSS Inc., Chicago, IL, USA), and *P* < 0.05 was considered statistically significant.

## Results

**Fig. 1:**
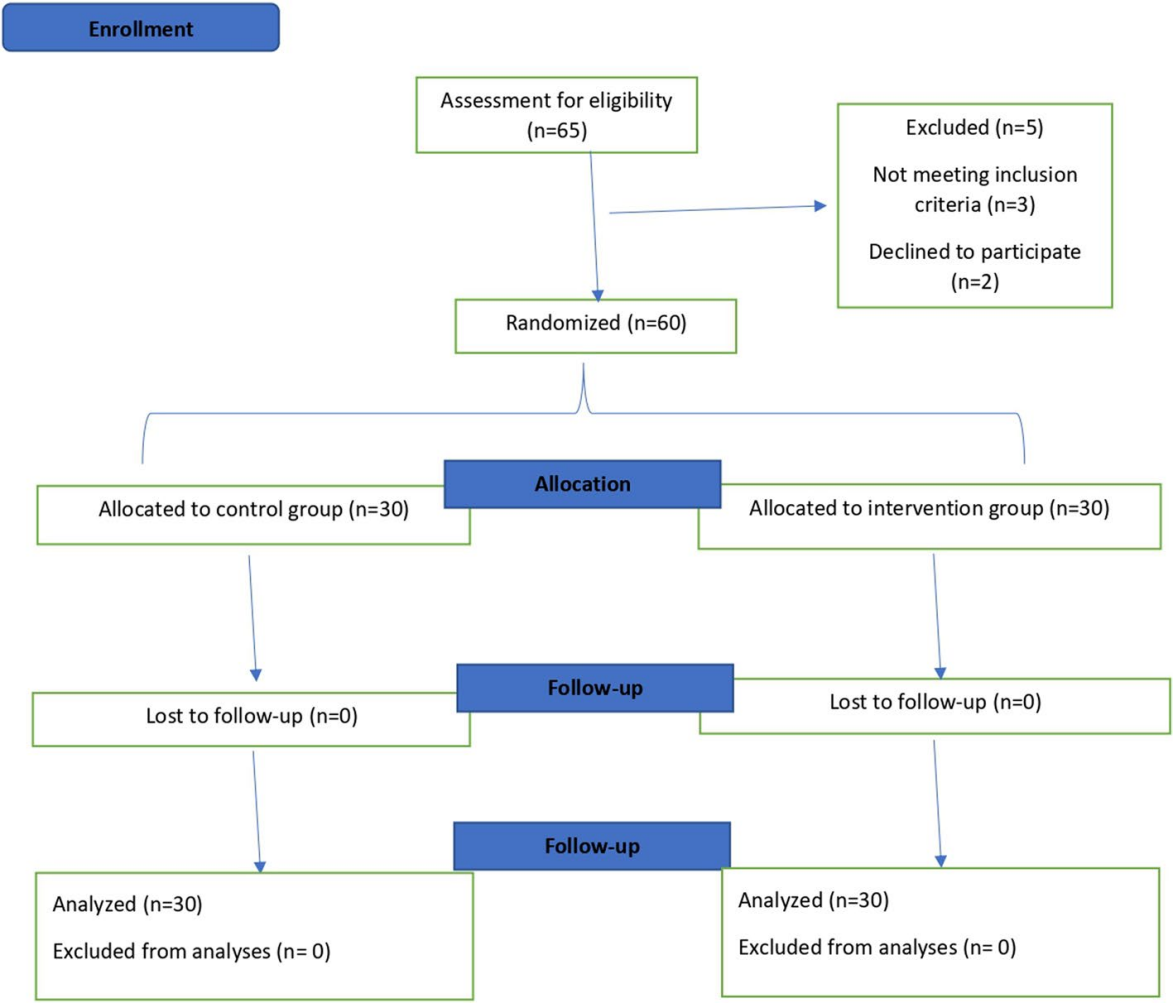
Flowchart of the trial

A total of sixty patients were enrolled in the study and randomly assigned to either the intervention group (*n* = 30) or the control group (*n* = 30) during July to August 2021 (Fig. 1). There were no significant differences between groups regarding general characteristics (Table 1). No side effects were reported by participants in either intervention group or control group following soup consumption.

**Table 1:** General characteristics of the participants in control group and intervention group at baseline

**Variable**	**Control group (*n* = 30)**	**Intervention group (*n* = 30)**	*P-value
**Age (years)**	50.33 ± 14.85	52.93 ± 13.40	0.48
**Weight (kg)**	82.60 ± 14.43	83.17 ± 13.97	0.88
**Body mass index (kg/m**^**2**^**)**	29.19 ± 4.14	29.61 ± 5.47	0.73
**Gender**			0.12
** Female**	19 (63.3)	13 (43.3)	
** Male**	11 (36.7)	17 (56.7)	
**Marital status**			0.98
**Single**	1 (3.3)	2 (6.7)	
**Married**	29 (96.7)	28 (93.3)	
**Education**			0.44
**Diploma and under diploma**	13 (43.3)	16 (53.3)	
**Above diploma**	17 (56.7)	14 (46.7)	

Data are presented as mean ± SD for quantitative and number (%) for qualitative variables

^*^Independent sample *t*-test was used for comparison of quantitative variables, and chi-square or Fisher’s exact tests were used for comparison of qualitative variables between the two groups

*P* < 0.05 was considered statistically significant

Table 2 demonstrates the results of blood biomarkers of participants before and after the intervention. The laboratory findings of the study groups were not significantly different before the intervention. The intervention group showed significant reductions in Na (*P* = 0.004) and SGOT (*P* < 0.001) levels and increases in K (*P* < 0.001) levels after a week of intervention. In control group, the D-dimer (*P* = 0.04) and BUN (*P* < 0.001) significantly increased, and SGOT (*P* = 0.03) and Na (*P* = 0.01) significantly decreased. Between-group comparisons between showed significant differences between the groups in terms of BUN (*P* = 0.01), Na (*P* = 0.04), and K (*P* = 0.03). After adjusting for confounders (baseline values, age, sex, and BMI), the soup containing functional food items was more effective in controlling serum levels of D-dimer (*P* = 0.01), BUN (*P* < 0.001), and Cr (*P* = 0.03). Further, K levels were significantly higher in the intervention group than in the control group (*P* = 0.03).

**Table 2:** The effects of functional food intervention on blood biomarkers

Variables	Control group (*n* = 30)	Intervention group (*n* = 30)	*P-value	^**^P-value	^***^P-value
D-dimer (mg/l)					
Baseline	0.87 ± 0.43	0.83 ± 0.44	0.75		
After intervention	1.68 ± 2.03	0.99 ± 0.23	0.07	0.08	0.01
****P-value	0.04	0.07			
BUN (mg/dl)					
Baseline	17.37 ± 12.57	17.74 ± 4.41	0.88		
After intervention	24.02 ± 11.26	17.36 ± 4.33	0.01	< 0.001	< 0.001
****P-value	< 0.001	0.69			
Cr (mg/dl)					
Baseline	1.27 ± 1.68	0.99 ± 0.12	0.37		
After intervention	1.24 ± 0.94	0.99 ± 0.11	0.16	0.04	0.03
****P-value	0.82	0.96			
SGOT (u/l)					
Baseline	38.11 ± 14.62	40.82 ± 18.16	0.53		
After intervention	30.55 ± 15.95	26.58 ± 3.15	0.19	0.12	0.05
****P-value	0.03	< 0.001			
SGPT (u/l)					
Baseline	40.19 ± 19.17	41.20 ± 21.08	0.85		
After intervention	41.90 ± 26.46	41.80 ± 13.53	0.98	0.90	0.92
****P-value	0.73	0.96			
LDH (u/l)					
Baseline	652.80 ± 146.90	696.36 ± 147.48	0.26		
After intervention	850.40 ± 66.06	670.45 ± 62.43	0.14	0.06	0.02
****P-value	0.08	0.38			
Na (mEq/L)					
Baseline	138.97 ± 2.64	137.68 ± 2.04	0.04		
After intervention	127.18 ± 24.57	136.57 ± 1.33	0.04	0.04	0.05
****P-value	0.01	0.004			
K (mEq/L)					
Baseline	3.88 ± 0.28	3.84 ± 0.21	0.57		
After intervention	3.90 ± 0.39	4.09 ± 0.22	0.03	0.03	0.04
****P-value	0.84	< 0.001			

*Abbreviations*: *BUN* blood urea nitrogen, *Cr* creatinine, *SGOT* serum glutamic-oxaloacetic transaminase, *SGPT* serum glutamic-pyruvic transaminase, *LDH* lactate dehydrogenase, *Na* sodium, *K* potassium

Values are expressed as means ± SD. *P* < 0.05 was considered as statistically significant

^*^Independent sample *t*-test was used for comparisons between groups at baseline and post-intervention

^**^Analysis of covariance (ANCOVA) was used for comparisons between the two groups post-intervention after adjusting for baseline values

^***^Analysis of covariance (ANCOVA) was used for comparisons between the two groups post-intervention after adjusting for baseline values plus other confounders such as age, sex, and BMI

^****^Paired *t*-test was used to compare within group difference from baseline to post-intervention period

*P* < 0.05 was considered statistically significant

In this study, the IL-1β, IL-6, IL-10, IL-17, CRP-q, TNF-α, and IFN-γ levels were also assessed and compared at baseline and post-intervention in both intervention and control groups (Table 3). At bassline, the results showed no significant differences between the two groups in terms of all factors. After intervention, the levels of IL-1β (*P* < 0.001), IL-6 (*P* < 0.001), IL-17 (*P* < 0.001), CRP-q (*P* < 0.001), TNF-α (*P* < 0.001), and IFN-γ (*P* < 0.001) were significantly decreased, and the levels of IL-10 (*P* < 0.001) significantly increased in both intervention and control groups. Between-group comparison showed greater improvement in intervention group in terms of IL-1β (*P* = 0.002), IL-6 (*P* < 0.001), IL-17 (*P* < 0.001), IL-10 (*P* < 0.001), and TNF-α (*P* < 0.001). In contrast, the control intervention reduced CRP-q more effectively than the functional soup group (*P* < 0.001). The results remained significant after adjustment for confounders (baseline values, age, sex, and BMI).

**Table 3:** The effects of functional food intervention on inflammatory markers

**Variables**	**Control group** **(*n* = 30)**	**Intervention group** **(*n* = 30)**	*P-value	^**^P-value	^***^P-value
IL-1β (pg/ml)					
Baseline	109.13 ± 13.11	115.84 ± 12.90	0.05		
After intervention	74.00 ± 12.49	63.81 ± 11.70	0.002	0.003	0.003
****P-value	< 0.001	< 0.001			
IL-6 (pg/ml)					
Baseline	76.82 ± 7.27	77.32 ± 8.45	0.80		
After intervention	54.45 ± 9.81	37.63 ± 6.77	< 0.001	< 0.001	< 0.001
****P-value	< 0.001	< 0.001			
IL-17 (pg/ml)					
Baseline	174.39 ± 6.99	174.68 ± 8.16	0.88		
After intervention	138.77 ± 4.78	128.71 ± 9.72	< 0.001	< 0.001	< 0.001
****P-value	< 0.001	< 0.001			
IL-10 (pg/ml)					
Baseline	34.21 ± 5.16	30.39 ± 6.09	0.01		
After intervention	61.05 ± 8.43	86.13 ± 5.11	< 0.001	< 0.001	< 0.001
****P-value	< 0.001	< 0.001			
CRP-q (mg/l)					
Baseline	23.17 ± 14.59	22.31 ± 13.46	0.81		
After intervention	8.12 ± 4.54	11.99 ± 6.15	0.001	0.003	0.01
****P-value	< 0.001	< 0.001			
TNF-a (pg/ml)					
Baseline	76.38 ± 7.58	75.66 ± 6.73	0.70		
After intervention	45.27 ± 8.70	7.98 ± 4.94	< 0.001	< 0.001	< 0.001
****P-value	< 0.001	< 0.001			
IFN-γ (pg/ml)					
Baseline	125.16 ± 8.74	120.30 ± 5.45	0.01		
After intervention	105.08 ± 13.87	103.96 ± 9.17	0.71	0.24	0.09
****P-value	< 0.001	< 0.001			

*Abbreviations*: *IL-1β* interleukin-1 beta, *IL-6* interleukin-6, *IL-17* interleukin-17, *IL-10* interleukin-10, *CRP-q* quantitative C-reactive-protein, *TNF-a* tumor necrosis factor-alpha, *IFN-γ* interferon-gamma

Values are expressed as means ± SD. *P* < 0.05 was considered as statistically significant

^*^Independent sample *t*-test was used for comparisons between groups at baseline and post-intervention

^*^*Analysis of covariance (ANCOVA) was used for comparisons between the two groups post-intervention after adjusting for baseline values

^***^Analysis of covariance (ANCOVA) was used for comparisons between the two groups post-intervention after adjusting for baseline values plus other confounders such as age, sex, and BMI

^****^Paired *t*-test was used to compare within group difference from baseline to post-intervention period

*P* < 0.05 was considered statistically significant

## Discussion

We conducted a double-blind, randomized controlled trial in COVID-19 patients to determine the effectiveness of a combination of functional foods on the serum levels of inflammatory cytokines and laboratory findings. Our research found beneficial effects of a soup containing functional foods in controlling serum levels of D-dimer, BUN, and Cr compared to a usual soup. The results also showed greater effectiveness of the functional foods containing soup in the improvement of some inflammatory markers, including IL-1β, IL-6, IL-17, IL-10, and TNF-α. Interesting to note, this study has found that soup containing functional foods has a beneficial effect on inflammatory markers as a dependent factor associated with COVID-19 disease severity [4, 5]. There is evidence that coronaviruses can lead to cytokine imbalances that damage a variety of organs [30]. Patients with severe COVID-19, as evidenced in our study, release a large amount of pro-inflammatory cytokines (IFN-α, IFN-γ, IL-1β, IL-6, IL-12, IL-18, IL-33, TNF- α, and TGF- β) [31]. It has been suggested that nuclear factor kappa B (NF-κB) is associated with inflammatory reactions, and blocking it will repress the transcription of the target genes, resulting in reduced inflammation [18]. We used a mixture of functional foods that included nutrient-rich compounds such as fruits, vegetables, nuts, grains, and beans. This combination was able to reduce inflammation in COVID-19 patients, which was probably due to the components of these items, vitamin C, vitamin E, vitamin A, beta-carotene, fiber, antioxidants, and curcumin [32].

Vitamin C has potent antioxidant, anti-inflammatory, and anti-viral features, which might improve general health conditions and thus help in the management of COVID-19 and its relevant complications like sepsis and pneumonia [33]. As part of the mechanism of vitamin C against COVID-19, this vitamin modulates activation of various immune cells, regulates the production of anti-inflammatory markers, and decreases histamine levels [34]. It can also raise the differentiation and proliferation of B and T cells and thus help the production of antibodies against SARS-CoV2 [35]. Another potent anti-inflammatory vitamin in our soup, vitamin E, suppresses the production of pro-inflammatory cytokines [36]. In addition, the powerful anti-inflammatory effect of vitamin A is caused by its ability to modulate and reduce inflammation in the body by elevating the production of T cells, natural killer cells, and neutrophils, thereby protecting the body from viral infections such as COVID-19 [37]. The soup containing functional soup also contains carotenoids, as the most important lipid-soluble phytochemicals with anti-inflammatory properties [38]. Several studies have shown that carotenoids can reduce NF-κB activation [39–42]. The most plentiful carotenoids in plasma include lutein, lycopene, and β-carotene [43]. Studies have shown that supplementation of 20 mg lutein daily for 3 months resulted in a reduction in plasma IL-6 compared with placebo in early arthrosis patients [44]. In addition, consumption of a tomato-based drink for 26 days lowered TNF-α secretion by 34% [45]. In contrast, it is reported that an intake of 2 or 8 servings of fruits and vegetables daily for 4 weeks did not show any impact on TNF-α, IL-12, and CRP; however, consuming more than 8 servings/day declined CRP concentration [46]. In our study, serum levels of inflammatory markers were reduced remarkably, which was possibly due to the synergistic effect of carotenoids with other micronutrients like vitamins.

Our soup also contains turmeric, a rich source of curcumin. It is a bright yellow chemical substance in plants that has been confirmed to have several properties, including anticancer, anti-inflammatory, anti-invasive, anti-angiogenic, immunomodulatory, and antioxidant activities in different diseases [47]. A previous meta-analysis showed that curcumin was able to reduce the amount of circulating IL-6 in patients with systemic inflammation [48]. Also, in another meta-analysis study, curcumin supplementation significantly improved TNF-α concentration [49]. It is supposed that curcumin blocks NF-κB and mitogen-activated protein kinase (MAPK) pathways, which are the essential signals regulating the expression of various pro-inflammatory cytokines [49]. The evidence also indicated the effect of curcumin on interferons in different viral diseases [50–52].

High intake of dietary fiber may also contribute to the beneficial effects of the soup containing functional foods. It has been suggested that dietary fiber may reduce the risk of some diseases with systemic inflammation by mediating the pro-inflammatory process [53–55]. These probable roles may be induced through two mechanistic hypotheses. First, dietary fiber may decrease the oxidation of glucose and lipids while maintaining a healthy intestinal environment [56]. Second, it is known that dietary fiber may reduce inflammation by modifying adipocytokines in adipose tissue and increasing the circulation of lipophilic compounds and lipids through the enterohepatic system [57]. In a study of postmenopausal women, greater intake of total fiber, soluble fiber, and insoluble fiber was related to lower plasma concentrations of IL-6 and TNF-α-R2 [19].

Finally, the beneficial effects of intervened soup might be because of its quercetin content. It is a widely distributed plant flavonoid (flavanol), usually found in fruit, vegetables, leaves, seeds, and grains, that has antioxidant, anti-inflammatory, ant-viral, and immunomodulatory activities [58, 59]. Quercetin showed to inhibit the production of various proinflammatory cytokines that have been proposed as the mechanism by which it acts against COVID-19 [60].

In this study, the aim was to introduce a natural mixture that would alleviate COVID-19-related complications. The study had randomized double-blind design which increases the power of the study. In addition, the analyses were controlled for potential confounders. However, the study had some limitations. This study had short duration and did not differentiate between participants with mild and moderate disease. Also, due to ethical consideration, we were unable to have a group without any intervention. Moreover, a number of clinical findings, particularly those associated with organ dysfunctions, were not assessed.

## Conclusion

This study explored potential effects of functional foods on the modulation of the inflammatory markers and laboratory findings in the COVID-19 patients. Accordingly, it indicated that functional foods mixture could modulate the secretion of serum inflammation markers efficiently. However, the results in terms of biochemical findings showed to be non-significant. Thus, further studies with longer duration are needed to confirmed the findings of this study.

## Data Availability

The dataset (s) supporting the conclusions of this article is available on reasonable request.
